# ApoE rs429358 and rs7412 Polymorphism and Gender Differences of Serum Lipid Profile and Cognition in Aging Chinese Population

**DOI:** 10.3389/fnagi.2017.00248

**Published:** 2017-08-02

**Authors:** Jie Zhen, Xiaochen Huang, Nicholas Van Halm-Lutterodt, Shengqi Dong, Weiwei Ma, Rong Xiao, Linhong Yuan

**Affiliations:** ^1^School of Public Health, Capital Medical University Beijing, China; ^2^Department of Neurosurgery, Beijing Tiantan Hospital, Capital Medical University Beijing, China

**Keywords:** apolipoprotein E, polymorphism, gender, lipid profile, cognitive function, geriatrics

## Abstract

ApoE gene polymorphism has been reportedly associated with serum lipids and cognition. However, very few studies have explored the combined effects of ApoE gene polymorphism and gender on serum lipid profile with subsequent impacts on cognition in Chinese population. A total of 1,000 Chinese community dwellers aged 55 years and above were recruited in this cross-sectional study. Demographic information of the participants was collected using well designed self-administered questionnaires. The Montreal Cognitive Assessment (MoCA) test was employed to evaluate the cognitive status of the participants. Semi-quantitative food frequency questionnaire (FFQ) was used to obtain the dietary intake information. Fasting venous blood samples were taken for ApoE genotyping and serum lipid measurements. Significant gender differences in cognition, serum lipid profile and dietary fat-rich foods consumption were observed (*p* < 0.05). Cognition of the subjects was found to be associated with ApoE genotypes (*p* < 0.05). ApoE rs429358 and rs7412 variants demonstrated a significant effect on cognitive performance in the male subjects; especially within the attention and language cognitive domains as well as the total MoCA score (*p* < 0.05), respectively. Serum lipid profile and cognition of Chinese adults are significantly linked with gender and ApoE genetic polymorphism. The ApoE variant rs429358 is found to be notably associated with cognition in aging male Chinese population.

## Introduction

Apolipoprotein E (apoE) is a multifunctional protein which transports and delivers cholesterol and other lipids in the plasma via binding to cell surface apoE receptors (Zhao et al., 2017). The human ApoE gene is polymorphic (derived from the combination of polymorphic rs429358 and rs7412), which results in 3 major isoforms (ε2, ε3, and ε4). Plasma lipids and lipoproteins are under strong genetic influence by the ApoE polymorphism (Egert et al., 2012). Carriers of the ε4 allele of the ApoE gene (ApoE ε4) have higher total and low-density lipoprotein cholesterol levels than non-carriers. Therefore, the ε4 allele is a strong genetic risk factor for heart disease in the general population (Reily et al., 1991; Rasmussen, 2016). Some published studies have demonstrated the ethnic differences of the existing relationship between ApoE genotypes and serum lipids profile (Smalinskiene et al., 2013; Jeenduang et al., 2015). These differences may also account for the relatively small percentage outcomes of the ApoE ε4 carriers in the Chinese population. Up to date, very few studies have explored the potential associations of ApoE rs429358 and rs7412 polymorphism with serum lipid profile in aging Chinese population. It is therefore necessary to explore the possible relationships between ApoE rs429358 and rs7412 polymorphism with lipid profile in aging Chinese adults.

Although ApoE was originally discovered and characterized for its role in plasma lipids metabolism, nowadays, it is recognized to have a major impact on neuronal function (Hauser et al., 2011). Except to expression of ApoE in liver and macrophages, ApoE was also expressed in brain, predominantly astrocytes and microglia. In brain, apoE is secreted as a lipid-poor protein that accrues lipid to form brain specific lipoprotein particles (Fan et al., 2009). The association of ApoE genetic polymorphism with Alzheimer's disease (AD) has also been addressed in epidemiological studies (Barrett et al., 2016) and experimental animal studies (Lannfelt and Nordstedt, 2000). Increased peripheral lipid levels and decreased cerebral glucose metabolism in the brain have been found in ApoE ε4 carriers (Agarwal and Tripathi, 2014). Additionally, ApoE protein has also been identified as an important key factor in affecting the pathogenesis of neurological diseases (Verghese et al., 2011). Studies comparing ApoE3 and ApoE4 have documented their differences in binding to Aβ (Holtzman et al., 2000; Tokuda et al., 2000). Such differences are entirely consistent with the revelation that ApoE4 is the major genetic risk factor for late-onset AD (Corder et al., 1993). The potential mechanisms of ApoE allele status on AD involves the aggregation and clearance of beta-amyloid (Aβ), modulation of neurotoxicity and tau phosphorylation, while affecting synaptic plasticity and neuro-inflammation (Cruts and Van Broeckhoven, 1998). More recently, ApoE gene polymorphism has been implicated with cognitive decline in healthy individuals (Chu et al., 2014). It is also generally known that the ApoE 4 allele is a derivative of rs429358 and rs7412 polymorphism. Given the linkage between ApoE genetic polymorphism and age-related cognitive decline in the elderly, it becomes critically necessary to explore the possible relationships between ApoE rs429358 and rs7412 polymorphism with cognitive function in older Chinese population.

Gender susceptibility to AD has been well documented in the literatures. Some previous studies have also indicated that, in regard to gender, there appears to be a rather higher prevalence of AD in women than observed in men (Fratiglioni et al., 1997; Andersen et al., 1999). The dramatic change of hormonal profile in women after menopause is suspected to be responsible for this outcome, and perhaps consequently exert strong effects on their health and overall quality of life. Moreover, a human-based clinical interventional study also discovered that the neuro-protective effects of hormones might rely on ApoE genotypes. Rippon et al. demonstrated that, during estrogen replacement therapy (ERT), there is a synergistic neuro-protective effect of estrogen with ApoE polymorphism in the ApoE ε4-negative carrier female population. In another cross-sectional study, ERT reduced the risk of familial AD by 80%, however, no association was found between ERT and the susceptibility to AD in female ApoE ε4 carriers (Rippon et al., 2006). All these results suggest that the ApoE gene may be a significant determinant and possible therapeutic target of female hormonal function and potency for the prevention of AD. Also, these data further hint out the modulating effects of ApoE genotype regarding the presumed related risk of gender-associated disparities with cognition decline and AD in the population.

Increasing evidence suggest the relationship between ApoE genetic polymorphism and gender with serum lipid profile and AD. Up to date, very few studies have reported on such combined effects of ApoE genetic polymorphism and gender on the serum lipid profile in association with cognitive function in aging Chinese adults. In present work, a cross-sectional study was designed to explore the possible relationships between ApoE gene polymorphism, gender and serum lipids with subsequent effects on cognition in older Chinese population. The objective of this study was to explore whether serum lipid profile and cognition were associated with gender in community-based aging adults. Furthermore, it becomes more interesting to explore whether the existing relationship between gender and serum lipids in regard to cognition in aging Chinese adults is dependent on ApoE polymorphism.

## Methods

### Participants

The study was a community-based cross-sectional study, and the design protocol was approved by the Human Ethics Committee of the Capital Medical University (No. 2012SY23). The procedures followed the ethical standards of the Helsinki Declaration of 1975. A total number of 1,000 community dwellers aged 55 years and above were randomly recruited by advertisements and direct phone dialing by the nurses from Nanyuan and Wulituo Community service centers, Beijing, China. The criteria for exclusion included uncontrolled diabetes mellitus, severe inflammatory conditions, recent history of heart or respiratory failure, chronic liver disease or renal failure, malignant tumors, and illness with poor prognosis. Subjects with conditions known to affect cognitive function (e.g., a recent history of alcohol abuse, history of cerebral apoplexy or cerebral infarction); as well as subjects with AD, Parkinson's disease (PD), long-term frequency intake of antidepressants and medication acting on central nervous system, and recently under lipid lowering medication treatment were also excluded from the present study. Written informed consent was obtained from all enrolled participants.

### Socio-demographic variables and anthropometric measurements

Anthropometric measures (height and weight) were documented by the nurses from the community medical service center. BMI was calculated as weight (kg)/height (m^2^). Educational level was assessed as the highest level attained and classified into six categories (illiterate, primary school, junior high school, high school, junior college, undergraduate and above). Information on demographic characteristics (gender, age), lifestyle factors [e.g., smoking (yes or no), alcohol drinking (never, 1–3 times/wk, 4–5 times/wk, >5 times/wk), physical activity (never, 1–3 times/wk, 4–5 times/wk, everyday)] was collected by using a well-structured self-administered questionnaire.

### Cognitive test

Cognitive function was assessed by Montreal Cognitive Assessment (MoCA), which consists of seven cognitive domains including visual-spatial and executive ability, naming, attention, abstraction, language, delayed memory recall and orientation functions. The test was carried out by trained investigators in the Nanyuan and Wulituo Community Health Service Center.

### Dietary assessment

Participants were visited at a community health service center by specifically trained nutritionists and registered nurses. A validated semi-quantitative food frequency questionnaire (FFQ) was used to assess the habitual consumption of 10 food groups (fruit and vegetable, whole grain, legume, red meat, poultry, fish, eggs, nuts, cooking oil, milk, comprising 35 items in total). This questionnaire was adopted from a questionnaire used for the Dietary Investigation of Chinese Residents, which was organized by the Chinese Nutrition Society (CNS) (Zhang et al., 2009). The food intake survey documented the information, including the consumption frequencies (daily and weekly) and the amount of foods consumed.

### DNA isolation and genotyping

Peripheral blood samples (6 ml intravenously) were collected in vacuum tubes and stored at −80 °C. DNA was extracted from frozen peripheral blood using the Wizare genomic DNA purification kit (Promega, Madison, WI, USA). ApoE genotypes were determined by Polymerase Chain Reaction (PCR) amplification and Restricted Fragment Length Polymorphism (RFLP) analysis according to the method described by Hixson (Hixson and Vernier, 1990). The specific primers used for ApoE genotyping are: forward, 5′-GGC ACG GCT GTCCAA GGA-3′; reverse, 5′-GCC CCG GCC TGG TAC ACT GCC-3′. For the purpose of quality control of the genotyping, 20% of DNA samples were dependably genotyped again by different operators.

### Serum parameter measurement

Blood samples were drawn after 12 hour (h) fasting. Then, centrifuged at 1,500 g for 15 min at 4 °C, serum was separated within 2 h, and all samples were stored at −40 °C until further laboratory tests. An ILAB600 clinical chemistry analyzer (Instrumentation Laboratory, Lexington, WI, USA) was used to determine serum total cholesterol (TC) and triglyceride (TG). High density lipoprotein cholesterol (HDL-C) was measured by using a commercially available assay from Instrumentation Laboratory (Lexington, WI, USA). Low density lipoprotein cholesterol (LDL-C) was calculated by using the Friedewald formula (Friedewald et al., 1972). All samples for each participant were analyzed within a single batch, and the inter-assay coefficients of variation (CV) were less than 5%.

### Statistical analyses

Data was analyzed with the software SPSS 19.0 (Chicago, IL, USA). Continuous variables were presented as mean (95% confidence interval, CI) or means ± standard deviation (SD). Gender, smoking, alcohol drinking, physical activity were presented as category variables. Participants were classified according to categories of ApoE rs429358, rs7412 and gender. General linear model (GLM) was used to compare the means of the detected parameters between the groups. Interaction between each variant and gender was examined to test genetic effects on differences in gender.

When comparing gender or ApoE genotype differences in cognition, confounding factors including age, gender, BMI, education, smoking, alcohol drinking, and physical activity were critically adjusted. When comparing gender or ApoE genotype differences in serum lipids, confounding factors including age, gender, BMI, smoking, alcohol drinking, and physical activity were also adjusted. *P* < 0.05 was considered to be statistically significant.

## Results

### Demographics of participants

Initially, a total of 1,000 aging Chinese adults participated in the present study. Eighty six subjects were excluded due to uncompleted questionnaires, unsuccessful biological specimen sampling or unsuccessful genotyping. After eliminating missing data, 914 subjects who satisfied the criteria were included for data analysis. As illustrated in Table 1, the mean age of the participants was 62.88 ± 5.72 years; 31.73 and 68.27% of the subjects were male and female, respectively. The mean BMI of the subjects was 25.50 ± 6.90 (kg/m^2^). 16.08% of the subjects reported to have the habit of smoking. 26.81% of the subjects were habitual alcohol consumers. Within the 914 subjects, only 7 subjects were of the ApoE rs429358 C/C genotype (accounting for 0.76% of all subjects). Only 10 subjects were detected with ApoE rs7412 T/T genotype (accounting for 1.09% of all subjects). Therefore, during the data analysis, the carriers of one or two copies of the C allele were pooled for ApoE rs429358 variant; and the carriers of one or two copies of the T allele were pooled for ApoE rs7412 variant. In total, ~83.1% of all subjects expressed the ApoE rs429358 T/T genotype, while ~84.4% subjects expressed the ApoE rs7412 C/C genotype. Significant gender difference of serum lipid profile was observed. Compared to the male subjects, the female subjects demonstrated a rather higher serum TC, TG, LDL-C and HDL-C levels (*p* < 0.05).

**Table 1:** Demographic characteristic of the participants.

**Demographic character**	**Gender**		**Total (*n* = 914)**	***P*-value**
	**Male (*n* = 290)**	**Female (*n* = 624)**		
Age, mean ± SD	63.6 ± 5.6	62.6 ± 5.7	62.9 ± 5.7	0.011
BMI (kg/m^2^), mean ± SD	25.1 ± 3.7	25.7 ± 8.0	25.5 ± 6.9	0.267
Education, n (%)				0.000
Illiterate	3 (1.0)	30 (4.8)	33 (3.6)	
Primary school	24 (8.3)	116 (18.6)	140 (15.3)	
Junior high school	151 (52.2)	276 (44.2)	427 (46.7)	
High school	75 (26.0)	159 (25.5)	234 (25.6)	
Junior college	22 (7.6)	30 (4.8)	52 (5.7)	
Undergraduate and above	15 (5.2)	13 (2.1)	28 (3.1)	
**LIFE STYLE**				
Smoking, n (yes, %)	120 (41.4)	27 (4.3)	147 (16.1)	0.000
Alcohol drinking, n (%)				0.000
Never	137 (47.2)	520 (83.3)	657 (71.9)	
1–3 times/week	85 (29.3)	78 (12.5)	163 (17.8)	
4–5 times/week	46 (15.9)	16 (2.6)	62 (6.8)	
>5 times/week	22 (7.6)	10 (1.6)	32 (3.5)	
Physical activity, n (%)				0.289
Never	30 (10.3)	58 (9.3)	88 (9.6)	
1–3 times/week	32 (11.0)	87 (13.9)	119 (13.0)	
4–5 times/week	43 (14.8)	70 (11.2)	113 (12.4)	
Everyday	185 (63.8)	409 (65.5)	594 (65.0)	
**GENOTYPE**				
ApoE rs429358				0.181
T/T, n (%)	234 (80.7)	526 (84.3)	760 (83.2)	
C/T, n (%)	56 (19.3)	98 (15.7)	154 (16.8)	
ApoE rs7412				0.767
C/C, n (%)	243 (83.8)	529 (84.8)	772 (84.5)	
C/T, n (%)	47 (16.2)	95 (15.2)	142 (15.5)	
**SERUM LIPIDS PROFILE**				
TC (mmol/L), mean ± SE	4.70 ± 0.06	5.21 ± 0.04	4.96 ± 0.05	0.000
TG (mmol/L), mean ± SE	1.68 ± 0.07	1.91 ± 0.06	1.84 ± 0.07	0.024
LDL-C (mmol/L), mean ± SE	2.95 ± 0.05	3.24 ± 0.04	3.11 ± 0.44	0.000
HDL-C (mmol/L), mean ± SE	1.27 ± 0.02	1.41 ± 0.01	1.33 ± 0.02	0.000

*BMI, body mass index; ApoE, Apolipoprotein E; TC, total cholesterol; TG, triglyceride; LDL-C, low density lipoprotein cholesterol; HDL-C, high density lipoprotein cholesterol*.

### Dietary intake according to gender

As shown in Table 2, male subjects reported to have much higher daily red meat, poultry and eggs intakes than the female subjects (*p* < 0.05). The female subjects reported to have higher amounts of fruit as well as fruit + vegetables intakes than the male subjects (*p* < 0.05).

**Table 2:** Dietary intakes according to gender in Chinese adults.

**Foods (g/d)**	**Male (*n* = 290)**	**Female (*n* = 624)**	***P*-value**
Fruit	139.61 (125.98, 153.24)	158.75 (149.88, 167.63)	0.000
Vegetable	318.05 (301.35, 334.76)	307.21 (296.34, 318.09)	0.735
Fruit + vegetable	457.66 (434.23, 481.10)	465.97 (450.71, 481.22)	0.010
Legume	36.84 (33.41, 40.28)	34.05 (31.82, 36.29)	0.775
Whole grain	37.30 (34.15, 40.45)	32.64 (30.59, 34.69)	0.820
Red meat	31.14 (27.64, 34.64)	22.56 (20.29, 24.83)	0.000
Poultry	21.35 (19.24, 23.46)	16.05 (14.6, 17.43)	0.000
Fish	29.04 (26.57, 31.51)	26.02 (24.41, 27.63)	0.050
Cooking oil	33.32 (30.98, 35.65)	31.93 (30.41, 33.45)	0.418
Milk	100.15 (89.18, 111.13)	94.59 (87.45, 101.74)	0.705
Egg	27.33 (25.29, 29.38)	23.41 (22.08, 24.74)	0.015
Nut	17.54 (15.47, 19.61)	14.62 (13.27, 15.97)	0.311

*Data were expressed as mean (95% CI). General Line Model (GLM) was used for data analysis. Factors including age, BMI, smoking habit and physical activity levels were adjusted. P-value < 0.05 was considered as significance*.

### Serum lipids according to ApoE genotype

The ApoE genotype difference of serum lipid profile was presented in Table 3. Comparing the subjects with ApoE rs7412 C/T genotype, serum lipid profile of subjects with ApoE rs7412 C/C genotype have lower serum TG, HDL-C levels and higher LDL-C level (*p* < 0.05). While, no significant association of ApoE rs429358 genotype with serum lipid levels was observed in these aging Chinese adults (*p* > 0.05).

**Table 3:** Serum parameters according to ApoE genotype in Chinese adults.

**Parameters**	**ApoE rs429358**		***P*-value**	**ApoE rs7412**		***P*-value**
	**(T/T) (*n* = 760)**	**(C/T) (*n* = 154)**		**(C/C) (*n* = 772)**	**(C/T) (*n* = 142)**	
TC (mmol/l)	5.018 (4.941, 5.094)	5.140 (4.969, 5.310)	0.202	5.025 (4.949, 5.102)	5.108 (4.931, 5.285)	0.402
TG (mmol/l)	1.812 (1.709, 1.914)	1.938 (1.710, 2.166)	0.323	1.765 (1.664, 1.866)	2.200 (1.964, 2.436)	0.001
LDL-C (mmol/l)	3.130 (3.065, 3.195)	3.230 (3.086, 3.374)	0.888	3.188 (3.124, 3.252)	2.923 (2.774, 3.072)	0.014
HDL-C(mmol/l)	1.366 (1.342, 1.390)	1.362 (1.308, 1.416)	0.217	1.354 (1.330, 1.377)	1.430 (1.374, 1.485)	0.001

*Data were expressed as mean (95% CI). General Line Model (GLM) was used for data analysis. Factors including sex, age, BMI, smoking, alcohol drinking and physical activity levels were adjusted. P-value < 0.05 was considered as significance.TC, total cholesterol; TG, triglyceride; LDL-C, low density lipoprotein cholesterol; HDL-C, high density lipoprotein cholesterol; ApoE, Apolipoprotein E*.

### Cognition according to gender

After adjustment of age, BMI, education levels, smoking, alcohol drinking and physical activity, gender difference of cognitive function was observed in the participants. As shown in Table 4, male subjects demonstrated a relatively higher attention and abstraction abilities than the females (*p* < 0.05). No statistical significance was detected on other cognitive domains as well as the total MoCA score between male and female subjects (*p* > 0.05).

**Table 4:** Cognition according to gender in Chinese adults.

**Cognition**	**Gender**		***P*-value**
	**Male (*n* = 290)**	**Female (*n* = 624)**	
Visual and executive	3.98 (3.83, 4.13)	3.88 (3.78, 3.97)	0.288
Naming	2.95 (2.90, 3.01)	2.89 (2.86, 2.92)	0.078
Attention	5.47 (5.23, 5.61)	5.28 (5.18, 5.37)	0.038
Language	2.23 (2.13, 2.34)	2.21 (2.14, 2.28)	0.739
Abstraction	1.74 (1.65, 1.83)	1.59 (1.53, 1.64)	0.007
Memory and delayed recall	2.97 (2.79, 3.16)	3.10 (2.98, 3.22)	0.272
orientation	5.82 (5.72, 5.93)	5.77 (5.71, 5.84)	0.450
MoCA score	25.51 (24.94, 26.08)	24.95 (24.58, 25.32)	0.131

*Data were expressed as mean (95% CI). General Line Model (GLM) was used for data analysis. Factors including age, BMI, education, smoking, alcohol drinking and physical activity levels were adjusted. P-value < 0.05 was considered as significance*.

### Serum lipids according to gender and ApoE polymorphism

As illustrated in Table 5, we observed significant combined effects of gender and ApoE rs429358 variant on serum lipid levels. Female subjects with ApoE rs429358 C/T genotype have the highest serum TC, LDL-C and HDL-C levels compared with subjects with ApoE rs429358 T/T genotype (*p* < 0.05).

**Table 5:** Serum parameters according to gender and ApoE rs429358 in Chinese adults.

**Parameters**	**Male (*****n*** = **290)**		**Female (*****n*** = **624)**		***P*-value**
	**ApoE rs429358 (T/T) (*n* = 234)**	**ApoE rs429358 (C/T) (*n* = 56)**	**ApoE rs429358 (T/T) (*n* = 526)**	**ApoE rs429358 (C/T) (*n* = 98)**	
TC (mmol/l)	4.593 (4.438, 4.747)	4.719 (4.421, 5.016)	5.214 (5.116, 5.311)	5.334 (5.121, 5.546)	0.000
TG (mmol/l)	1.618 (1.411, 1.824)	1.916 (1.518, 2.313)	1.901 (1.770, 2.031)	1.941 (1.657, 2.225)	0.144
LDL-C (mmol/l)	2.881 (2.750, 3.012)	2.945 (2.694, 3.196)	3.245 (3.163, 3.327)	3.362 (3.183, 3.542)	0.000
HDL-C (mmol/l)	1.261 (1.212, 1.309)	1.242 (1.149, 1.336)	1.415 (1.384, 1.446)	1.418 (1.351, 1.485)	0.000

*Data were expressed as mean ± SE. General Line Model (GLM) was used for data analysis. Factors including age, BMI, smoking, alcohol drinking and physical activity levels were adjusted. P-value < 0.05 was considered as significance. TC, total cholesterol; TG, triglyceride; LDL-C, low density lipoprotein cholesterol; HDL-C, high density lipoprotein cholesterol; ApoE, Apolipoprotein E*.

As shown in Table 6, statistical significance of gender and ApoE rs7412 genotypes difference of the entirely studied lipid panel was observed. The highest serum TC, TG and HDL-C concentrations were observed in the female subjects with ApoE rs7412 C/T genotype (*p* < 0.05). While, the variant of ApoE rs7412 significant decreased serum LDL-C levels in both male and female subjects (*p* < 0.05).

**Table 6:** Serum parameters according to gender and APOE rs 7412 in Chinese adults.

**Parameters**	**Male (*****n*** = **290)**		**Female (*****n*** = **624)**		***P*-value**
	**APOE rs7412 (C/C) (*n* = 243)**	**APOE rs7412 (C/T) (*n* = 47)**	**APOE rs7412 (C/C) (*n* = 529)**	**APOE rs7412 (C/T) (*n* = 95)**	
TC (mmol/l)	4.597 (4.446, 4.749)	4.727 (4.412, 5.042)	5.223 (5.126, 5.320)	5.282 (5.063, 5.501)	0.000
TG (mmol/l)	1.662 (1.461, 1.862)	1.773 (1.355, 2.192)	1.813 (1.684, 1.942)	2.409 (2.188, 2.700)	0.001
LDL-C (mmol/l)	2.910 (2.783, 3.037)	2.792 (2.527, 3.056)	3.317 (3.235, 3.398)	2.978 (2.794, 3.162)	0.000
HDL-C (mmol/l)	1.239 (1.191, 1.286)	1.356 (1.258, 1.455)	1.407 (1.376, 1.437)	1.462 (1.393, 1.530)	0.000

*Data were expressed as mean ± SE. General Line Model (GLM) was used for data analysis. Factors including age, BMI, smoking, alcohol drinking and physical activity levels were adjusted. P-value < 0.05 was considered as significance. TC, total cholesterol; TG, triglyceride; LDL-C, low density lipoprotein cholesterol; HDL-C, high density lipoprotein cholesterol; ApoE, Apolipoprotein E*.

### Cognition according to ApoE genetic polymorphism

As shown in Table 7, ApoE rs429358 genetic variant significantly affected naming and orientation ability in aging Chinese adults (*p* < 0.05). However, there were no other significant ApoE rs429358 genotypic differences in other cognitive domains as well as total MoCA score outcomes (*p* > 0.05). No significant findings were observed between subjects with different ApoE rs7412 genotypes (*p* < 0.05).

**Table 7:** Cognition according to ApoE genotype in Chinese adults.

**Cognitive ability**	**APOE rs429358**		***P*-value**	**APOE rs7412**		***P*-value**
	**(T/T) (*n* = 760)**	**(C/T) (*n* = 154)**		**(C/C) (*n* = 772)**	**(C/T) (*n* = 142)**	
Visual and executive	3.92 (3.84, 4.01)	3.80 (3.62, 3.99)	0.244	3.93 (3.85, 4.01)	3.76 (3.57, 3.95)	0.108
Naming	2.92 (2.89, 2.95)	2.83 (2.76, 2.90)	0.015	2.91 (2.88, 2.94)	2.89 (2.82, 2.96)	0.563
Attention	5.34 (5.26, 5.42)	5.31 (5.13, 5.49)	0.803	5.36 (5.28, 5.44)	5.21 (5.02, 5.40)	0.158
Language	2.24 (2.18, 2.30)	2.11 (1.98, 2.24)	0.086	2.23 (2.17, 2.29)	2.16 (2.02, 2.29)	0.350
Abstraction	1.65 (1.60, 1.70)	1.59 (1.48, 1.70)	0.359	1.65 (1.60, 1.70)	1.59 (1.48, 1.70)	0.358
Memory and delayed recall	3.09 (2.99, 3.20)	2.91 (2.68, 3.13)	0.145	3.09 (2.99, 3.19)	2.91 (2.67, 3.14)	0.165
Orientation	5.81 (5.75, 5.87)	5.65 (5.52, 5.78)	0.029	5.79 (5.73, 5.85)	5.76 (5.62, 5.90)	0.685
MoCA score	25.25 (24.93, 25.57)	24.50 (23.79, 25.21)	0.060	25.23 (24.91, 25.54)	24.52 (23.77, 25.26)	0.087

*Data were expressed as mean (95% CI). General Line Model (GLM) was used for data analysis. Factors including sex, age, BMI, education, smoking, alcohol drinking and physical activity levels were adjusted. P-value < 0.05 was considered as significance. ApoE, Apolipoprotein E*.

### Gender and ApoE genotype differences with cognition

Significant combined effects of gender and ApoE genotypes on cognitive function were found in the current study. Gender and ApoE rs7412 genetic variant rather seem to mainly relate to the language ability in aging Chinese adults. The highest language ability was observed in the female subjects with ApoE rs7412 C/T genotype; while, the lowest language ability was observed in male subjects with ApoE rs7412 C/T genotype (*p* < 0.05) (Table 8). As demonstrated in Table 9, the lowest attention and language abilities as well as total MoCA score was observed in male subjects carrying the ApoE rs429358 C/T genotype (*p* < 0.05).

**Table 8:** Cognition according to gender and APOE rs7412 in Chinese adults.

**Cognition ability**	**Male*****(n** = **290)***		**Female(*n* =** ***624)***		***P*-value**
	**APOE rs7412 (C/C) (*n* = 243)**	**APOE rs7412 (C/T) (*n* = 47)**	**APOE rs7412 (C/C) (*n* = 529)**	**APOE rs7412 (C/T) (*n* = 95)**	
Visual and executive	3.90 (3.71, 4.10)	3.66 (3.16, 4.16)	3.90 (3.76, 4.03)	3.70 (3.31, 4.09)	0.785
Naming	2.91 (2.84, 2.99)	2.99 (2.81, 3.18)	2.86 (2.81, 2.91)	2.86 (2.71, 3.00)	0.534
Attention	5.50 (5.32, 5.69)	4.86 (4.37, 5.34)	5.29 (5.16, 5.42)	5.26 (4.87, 5.64)	0.054
Language	2.23 (2.09, 2.37)	1.77 (1.42, 2.12)	2.17 (2.07, 2.26)	2.25 (1.97, 2.53)	0.026
Abstraction	1.75 (1.64, 1.87)	1.72 (1.42, 2.01)	1.57 (1.49, 1.65)	1.51 (1.28, 1.74)	0.967
Memory and Delayed Recall	2.93 (2.69, 3.17)	2.51 (1.89, 3.12)	3.12 (2.95, 3.28)	2.72 (2.24, 3.21)	0.987
orientation	5.77 (5.63, 5.90)	5.58 (5.23, 5.93)	5.72 (5.63, 5.82)	5.77 (5.50, 6.05)	0.293
MoCA score	25.32 (24.58, 26.07)	23.47 (21.54, 25.39)	24.87 (24.35, 25.39)	24.32 (22.80, 25.84)	0.274

*Data were expressed as mean ± SE. General Line Model (GLM) was used for data analysis. Factors including age, BMI, education, smoking, alcohol drinking and physical activity levels were adjusted. P-value < 0.05 was considered as significance. ApoE, Apolipoprotein E*.

**Table 9:** Cognition according to gender and APOE rs429358 in Chinese adults.

**Cognition ability**	**Male (*****n*** = **290)**		**Female (*****n*** = **624)**		***P*-value**
	**ApoE rs429358 (T/T) (*n* = 234)**	**ApoE rs429358 (C/T) (*n* = 56)**	**ApoE rs429358 (T/T) (*n* = 526)**	**ApoE rs429358 (C/T) (*n* = 98)**	
Viso and executive	4.00 (3.79, 4.21)	3.57 (3.08, 4.06)	3.79 (3.65, 3.93)	3.80 (3.41, 4.20)	0.172
Naming	2.97 (2.89, 3.05)	2.94 (2.76, 3.12)	2.88 (2.83, 2.93)	2.83 (2.69, 2.98)	0.900
Attention	5.51 (5.30, 5.71)	4.85 (4.37, 5.33)	5.20 (5.06, 5.33)	5.35 (4.97, 5.73)	0.013
Language	2.24 (2.09, 2.39)	1.77 (1.42, 2.12)	2.19 (2.09, 2.29)	2.22 (1.95, 2.50)	0.031
Abstraction	1.76 (1.63, 1.88)	1.71 (1.42, 2.00)	1.56 (1.48, 1.64)	1.52 (1.28, 1.75)	0.935
Memory and delayed recall	2.98 (2.72, 3.24)	2.45 (1.84, 3.06)	3.09 (2.92, 3.26)	2.75 (2.27, 3.23)	0.545
orientation	5.90 (5.75, 6.05)	5.45 (5.10, 5.79)	5.75 (5.66, 5.85)	5.74 (5.46, 6.02)	0.061
MoCA score	25.72 (24.90, 26.53)	23.07 (21.18, 24.97)	24.67 (24.13, 25.20)	24.53 (23.01, 26.04)	0.042

*Data were expressed as mean ± SE. General Line Model (GLM) was used for data analysis. Factors including age, BMI, education, smoking, alcohol drinking and physical activity levels were adjusted. P-value < 0.05 was considered as significance. ApoE, Apolipoprotein E*.

## Discussion

Lipid metabolism remains a very essential part of nutrition and health since it is generally acknowledged that dietary fats and oils are important determinants of serum lipid profile (Howell et al., 1997). ApoE has been appreciated to execute significant roles in cholesterol transport and clearance in the central nervous system (CNS). Increasing evidences indicated the relationship between apoE and the pathophysiology of AD. ApoE4 contributes to AD pathogenesis by modulating multiple pathways, including the metabolism, aggregation, and toxicity of Aβ, tauopathy, synaptic plasticity and lipid transport (Verghese et al., 2011). Recently, the interactions between gender and ApoE in the pathological development of AD were indicated in experimental animal and human-based studies (Barrett-Connor and Goodman-Gruen, 1999; Caselli, 2012).

In this cross-sectional study, we observed significant gender differences of age, educational level, lifestyle, dietary intakes, serum lipid levels and cognitive function in aging Chinese population (Table 1). We adjusted the potentially confounding variables when analyzing serum lipid parameters and cognition in the male and female subjects by employing the general linear stats model (GLM). Higher serum lipid levels were detected in the female compared to the male subjects. This finding shows consistency with the results from previously reported studies (Swai et al., 2009; Ghobadzadeh et al., 2015). Anagnostis et al. observed in their study that the female subjects demonstrated a rather higher plasma TC and LDL-C levels than the male subjects (Anagnostis et al., 2015). Aging was suggested a possible factor that contributed to the observed gender differences in the serum lipid profile. Carroll et al. also reported that male subjects aged from (30 to 49) years demonstrated higher serum TC levels than female subjects; however, the female subjects notably showed a higher serum TC levels after the age 60 years (Carroll et al., 1993). Age-induced metabolic changes in gender may also be a contributory factor to the observed differences in the serum lipid patterns between the male and female subjects, which further reproduces the evidence of reciprocal TC levels' change observed as lower in female subjects prior to the age 60 with a dramatic increase after 60 years of age (Research Committee on Serum Lipid Level Survey, 1990).

After the adjustment of BMI and physical activity data, we observed a significant difference in dietary intake of male and female subjects. As illustrated in Table 2, female subjects consumed less fat-containing foods (such as red meat, poultry and eggs) and more fruits as well as total fruits + vegetables than the male subjects. However, their serum lipids were significantly higher compared to that observed in the male subjects (Table 1). These results hint that dietary consumption of fat-containing foods does not necessarily reflect the vivo lipid profile in aging male and female adults. It becomes more intriguing to observe differences in gender patterns in previously reported studies (Swai et al., 2009; Anagnostis et al., 2015; Ghobadzadeh et al., 2015; Mongraw-Chaffin et al., 2015) which correspond with the findings observed in our current study. With the exception of ApoE polymorphic variants, the reported consumption of fat-containing foods by participants did not tally with the outcomes of serum lipid levels in male and female subjects and therefore could not fully explain the impacts of diet on serum lipid levels and subsequently on nutritional intake gender-based cognitive outcomes in these older Chinese adults. Reasons for the observed serum lipid patterns may be attributed to age-induced metabolic changes, genetic differences and perhaps female gender role-play. It is therefore critically important to explore in further research, the possible reasons for such provocative patterns in the lipid panel of male and female subjects so as to formulate and provide guidance for dietary-nutrients intake for better metabolic and health outcomes in the elderly.

Genes involved in metabolic pathways have also been ascertained to contribute to serum lipid level variability. The knowledge of the role of ApoE genetic polymorphism in affecting different individual variations with plasma cholesterol especially, low-density lipoproteins (LDL-C) levels in the general population is also well established (Bennet et al., 2007; Willer et al., 2008). Radwan et al.'s study indicates that ApoE rs429358 and ApoE rs7412 polymorphism are significantly associated with plasma LDL-C levels (Radwan et al., 2014). In this present study, findings of significant statistical associations between genetic variation in ApoE rs7412 and serum LDL-C levels was established. Adults carrying the T allele of ApoE rs7412 demonstrated lower serum LDL-C levels. This result is consistent with previously reported studies (Bennet et al., 2010; Barbosa et al., 2012; Radwan et al., 2014). However, few studies have explored the impact(s) of ApoE polymorphism on plasma high-density lipoprotein (HDL-C) and triglycerides (TG) levels. In the present study, we also observed an ApoE genotype difference in serum TG and HDL-C levels. The subjects with ApoE rs7412 C/T genotype demonstrated highest serum TG and HDL-C levels (Table 3). However, carriers of the rs429358 variant of ApoE did not demonstrate any significant finding between ApoE rs429358 polymorphic types and serum lipids levels. These results may indicate that the impact of ApoE genetic polymorphism on serum lipids is possibly attributed to ApoE rs7412 C/T variant in older Chinese adults since no significance difference in lipid panel was found with carriers of the rs429358 variant.

The variants ApoE rs429358 and ApoE rs7412 were also reportedly associated with increased HDL-C and TG levels even though the investigators did not find any association with gender (Teslovich et al., 2010; Willer et al., 2013). Those results were partly consistent with our findings in the sense of revealing significant associations of ApoE's common variants (ApoE rs429358 and rs7412) with serum density-defined lipoproteins (HDL-C/LDL-C) and triglycerides (TG) (Table 3). In this study, we also observed unique patterns in gender and ApoE polymorphism with concomitant cognitive associations in aging Chinese population sample (Table 4). The association of ApoE genotype and serum lipids profile with gender has been implied in some publications (Dallongeville et al., 1992; Schaefer et al., 1994; Gomez-Coronado et al., 1999; Huang et al., 2006; Bennet et al., 2007; Tejedor et al., 2014). Gene-involved in distinct lipid traits (e.g., abnormal TG or HDL-C levels) have been identified in gender-based genome wide association study (Aulchenko et al., 2009). Katerina et al. discovered that ApoE isoforms and menopause may act as strong modulators in the levels of serum lipid profile (Katerina et al., 2010). In this current study, there appears to be a significant increase in female lipid profile respectively across the entire ApoE genotypes with gender taken into consideration (Tables 5, 6) in the studied population with the exception of TG carriers of ApoE rs429358 genotype (Table 5). It is reported that heredity may explain ~35–60% of variability in the plasma lipids (Weiss et al., 2006), which may suggest that, regardless of the genetic factors; other factors (perhaps including *in vivo* endocrine activities) may contribute to the gender variation of serum lipids traits in Chinese adults (Sertic et al., 2009). The inconsistency with our present study in comparison other reported studies might be attributed to the geographic and ethnic differences in different population studies as well as the physiological status of the participants.

Growing amounts of evidence suggest that gender difference is implicated in the incidence of AD with evidence showing that women seem to have a rather higher incidence risk of dementia than men (Ott et al., 1996; Ropacki and Jeste, 2005; Zuidema et al., 2009). Gender-specific neuro-psychiatric symptoms were also proven to be a generalized phenomenon in AD. Some studies have reported that male AD patients were more frequent to exhibit apathy and anxiety while female AD patients most often experienced delusion as a frequent and common neuro-psychiatric symptom. Based on these literature findings, we further explored whether there existed a gender difference in the cognition of these community elderly subjects. After the linear model adjustment for age, BMI, education, smoking, alcohol drinking and physical activity, we found significant gender differences in domains of attention and abstraction abilities (Table 4). Our results suggested a better cognitive performance of male than female subjects in these domains. As a neuron-protective factor, female hormones have been regarded to exert strong effects in keeping the normal function of the nervous system (Bielawska-Batorowicz et al., 2003; Bojar et al., 2013). It is also presumed that after the fallen hormone levels often appreciated during post-menopausal period, the decrease in female serum estrogen levels *in vivo* may perhaps play a role in the incidence of cognitive decline and psychological disorders in the female subjects (McCarthy, 2008). Future studies are therefore encouraged to aim at evaluating the effects of gender-related hormones to help provide a better understanding of the possibly complex existing relationship(s) between gender and cognition.

We also found that the ApoE genetic variants were cognition-associated in the elderly. ApoE rs429358 variant carriers expressed significant decrease in naming and orientation abilities in the overall studied population (Table 7). Our work is first of a kind with this outcome of ApoE variant rs429358 exerting such declination effects in some cognitive domains compared with a recent publication. Prada and colleagues reported a rather protective effect of only ApoE rs429358 variant on cognitive function (Prada et al., 2014); however, in our study, no association between ApoE rs7412 polymorphism with cognitive function was observed in the elderly which is rather suggestive that carriers of ApoE rs429358 variant are more susceptible to cognitive decline while on the contrary, carriers of ApoE rs7412 variant apparently seem to express a stronger resistance to cognitive decline compared to ApoE rs429358 variants in these aging Chinese population. With the exception of genetic factors, we speculate that, other gene-environmental factors associated with race and ethnic backgrounds might contribute to these observed inconsistent findings in differently reported studies. Furthermore, we detected a relationship between gender, ApoE genetic polymorphism and cognition. After the genotypes were further categorized by gender, it became quite intriguing to find out that both ApoE rs429358 and rs7412 variants seemed to express a reserved cognitive function in female gender. This is evidently demonstrated by the significantly higher language and attention ability functional domains in the female subjects with ApoE rs429358 C/T and/or rs7412 C/T genotypes (*p* < 0.05; Tables 8, 9). Conversely, the variant of ApoE rs429358 C/T genotype male carriers respectively demonstrated significant decline in attention and language abilities as well as the total MoCA score in the male subjects (*p* < 0.05; Tables 8, 9). Comprehensively, our data indicate pertinent combined effects of gender and ApoE rs429358 variant on cognition, especially on attention and language domains and overall cognitive functional ability in male Chinese adults.

In addressing the limitations associated with this present study, it is clear that this study is a cross-sectional design with a relatively small sample size and therefore to some extent hinders our ability to draw major conclusions on all our findings. In addition, the study was carried out in a sampled Chinese population; the variety of genotypic frequencies of ApoE gene in different populations around the world should be taken into consideration and also may possibly limit the extrapolations of our findings in regard to other ethnic populations. Finally, the gender difference in cognition might be related to gender-based *in vivo* endocrine activities. It has been reported that in the elderly, change of *in vivo* sex-hormone levels following aging might contribute to the decline in cognitive function in the elderly (Barrett-Connor and Goodman-Gruen, 1999; Vermeulen, 2001; Yaffe et al., 2002). In current study, we did not detect serum hormonal levels; as a result, we were unable to provide hormonal biomarker-based evidence to vividly elucidate the underlying associations of gender and ApoE genotypes with lipids profile and cognition in these aging Chinese adults. It is therefore encouraged that further large scale population based studies are conducted in the future to assist in elucidating the interplay between gender, ApoE genotype, lipid profile, cognition as well as hormones.

## Conclusion

In summary, this is a premier comprehensive study that evaluates the associations of gender, ApoE rs429358 and rs7412 polymorphism with serum lipids and impacts on cognition in aging Chinese population. Cognition and serum lipid profile are gender and ApoE rs429358 and rs7412 polymorphism associated. After considering the exception of diet, some other factors that deem to be correlated with ApoE gene expression may seem to contribute to the observed gender differences in serum lipid profile and cognition in aging Chinese adults.

## Author contributions

LY designed the work; JZ, XH, and SD carried out the questionnaire survey and collected the data; JZ and XH contributed to the lab work; LY, NV, and RX contributed to the data interpretation and drafting the manuscript; WM and SD did the statistical analysis.

### Conflict of interest statement

The authors declare that the research was conducted in the absence of any commercial or financial relationships that could be construed as a potential conflict of interest.
